# Cyclophilin B facilitates the replication of Orf virus

**DOI:** 10.1186/s12985-017-0781-x

**Published:** 2017-06-15

**Authors:** Kui Zhao, Jida Li, Wenqi He, Deguang Song, Ximu Zhang, Di Zhang, Yanlong Zhou, Feng Gao

**Affiliations:** 1 College of Veterinary Medicine, Jilin University, 5333 Xi’an Road, Changchun, 130062 China; 2 College of Public Hygiene, ZunYi Medical University, 201 Dalian Road, Zunyi, 563003 China; 3 Laboratory Animal Center, Peking University, 5 Summer palace Road, Beijing, 100871 China; 4 Key Laboratory of Zoonosis, Ministry of Education, College of Veterinary Medicine, Jilin University, 5333 Xi’an Road, Changchun, 130062 China

**Keywords:** ORFV, Cellular cyclophilin B, Cyclosporine A, RNA interference, Replication

## Abstract

**Background:**

Viruses interact with host cellular factors to construct a more favourable environment for their efficient replication. Expression of cyclophilin B (CypB), a cellular peptidyl-prolyl *cis-trans* isomerase (PPIase), was found to be significantly up-regulated. Recently, a number of studies have shown that CypB is important in the replication of several viruses, including Japanese encephalitis virus (JEV), hepatitis C virus (HCV) and human papillomavirus type 16 (HPV 16). However, the function of cellular CypB in ORFV replication has not yet been explored.

**Methods:**

Suppression subtractive hybridization (SSH) technique was applied to identify genes differentially expressed in the ORFV-infected MDBK cells at an early phase of infection. Cellular CypB was confirmed to be significantly up-regulated by quantitative reverse transcription-PCR (qRT-PCR) analysis and Western blotting. The role of CypB in ORFV infection was further determined using Cyclosporin A (CsA) and RNA interference (RNAi). Effect of CypB gene silencing on ORFV replication by 50% tissue culture infectious dose (TCID_50_) assay and qRT-PCR detection.

**Results:**

In the present study, CypB was found to be significantly up-regulated in the ORFV-infected MDBK cells at an early phase of infection. Cyclosporin A (CsA) exhibited suppressive effects on ORFV replication through the inhibition of CypB. Silencing of CypB gene inhibited the replication of ORFV in MDBK cells. In conclusion, these data suggest that CypB is critical for the efficient replication of the ORFV genome.

**Conclusions:**

Cellular CypB was confirmed to be significantly up-regulated in the ORFV-infected MDBK cells at an early phase of infection, which could effectively facilitate the replication of ORFV.

## Background

Orf virus (ORFV) is the type species of the genus *Parapoxvirus*, which has a worldwide distribution and is the causative agent of Orf, a contagious debilitating skin disease of sheep and goats also known as contagious ecthyma, contagious pustular dermatitis, infectious labial dermatitis, scabby mouth or sore mouth [1]. Primary infections are usually resolved within 1–2 months, however repeated and persistent infections can occur in the presence of an intensive inflammatory host antivirus immune response [2, 3]. The mechanisms that establish the repeated and persistent infections in vivo are almost completely unknown. Currently, the host immune response to ORFV has been extensively studied, yet many aspects of the complex host-virus interactions remain unclear.

Cyclophilins (Cyp) comprise a family of peptidyl-prolyl *cis/trans* isomerases, which are originally discovered as a cellular factor with high affinity for the immunosuppressant CsA [4, 5]. CypB is one of the most abundant members among the Cyp family, is ubiquitously expressed in most cells, and predominantly resides in the endoplasmic reticulum (ER) through the ER retention signal sequence in the C-terminus [6, 7]. CypB functions in various cellular processes, including transcriptional regulation, protein secretion, immune response and apoptosis [8–10]. In addition, some researchers have shown that many viruses such as Japanese encephalitis virus (JEV), hepatitis C virus (HCV) and human papillomavirus type 16 (HPV 16) require CypB for their replication [11–13]. However, there has been no report on the involvement of CypB in the replication of ORFV.

In the present study, CypB was found to be significantly up-regulated in the ORFV-infected MDBK cells at an early phase of infection. However, the function of cellular CypB in ORFV replication has not yet been explored. Sequently, we investigated the role of CypB in the replication of ORFV in MDBK cells. Cyclosporin A (CsA) exhibited suppressive effects on ORFV replication through the inhibition of CypB. Silencing of CypB gene inhibited the replication of ORFV in MDBK cells. In conclusion, these data suggest that CypB is critical for the efficient replication of the ORFV genome.

## Methods

### Cells and virus

The Madin―Darby bovine kidney (MDBK) cells were maintained in minimal essential medium (MEM) (GIBCO, Invitrogen), supplemented with 10% fetal bovine serum (FBS) and penicillin (100 U/mL); streptomycin (100 mg/mL); and nystatin (20 mg/mL). The Orf virus used in this study (ORFV-Jilin) was isolated using MDBK cells from scab specimens collected from skin lesions of a 6-week-old small-tailed Han sheep afflicted with orf in November 2008 in the Jilin province of China [14].

### Antibodies and reagents

The antibodies used in this study were anti-Cyclophilin B polyconal rabbit antibody (Abcam), PE-Cy5 conjugated rabbit anti-Cyclophilin B antibody (Bioss), anti-β-actin (Proteintech), Peroxidase-conjugated Affinipure Goat Anti-Mouse IgG (Proteintech) and Peroxidase-conjugated Affinipure Goat Anti-Rabbit IgG (Proteintech). CsA were purchased from Sigma. PolyATtract® mRNA Isolation Systems was purchased from Promega. PCR-Select™ cDNA Subtraction Kit and Advantage cDNA PCR Kit & Polymerase Mix were obtained from Clontech. X-treme GENE HP DNA Transfection Reagent and X-treme GENE siRNA Transfection Reagent were purchased from Roche.

### Virus infection

MDBK cells have been reported to be least partially permissive for ORFV replication [14]. A confluent monolayer of MDBK cells cultured in 75-cm^2^ flasks were adsorbed with ORFV-Jilin (multiplicity of infection [MOI] = 3) for 2 h at 37 °C. After adsorption, unbound virus was removed by gentle washing with serum-free medium followed by the addition of fresh medium and further incubation at 37 °C. At 2 h after further incubation, ORFV transcripts were detected in MDBK cells infected with ORFV-Jilin. Thus, the starting point of early phase of ORFV infection was determined for 4 h. The cultures of incubated cells were harvested at 4 h post infection, and cell lysates were collected. Normal cell controls were collected in a similar manner. All samples were frozen in liquid nitrogen and stored at −80 °C until SSH library construction.

### Construction and screening of Suppression subtractive hybridization (SSH) library

Total RNA was isolated from both mock- and ORFV-inoculated cell lysates using RNAiso Plus (Takara, Dalian, China) according to the manufacturer’s instructions. The quality of total RNA were determined by ultraviolet spectrophotometry (260 and 280 nm) and 1% agarose/ethidium bromide (EtBr) gel electrophoresis. Messenger RNA (mRNA) was isolated from total RNA using PolyATtract® mRNA Isolation Systems (Promega) according to the manufacturer’s instructions. The purified mRNA was treated with 1 mL of 75% ethanol and 0.1 volume of 3 mol/L NaOAc and used as the starting material to construct the SSH cDNA library. PCR select cDNA subtraction was conducted using PCR-Select™ cDNA Subtraction Kit (Clontech, USA) with 2 μg of mRNA for each sample as starting material according to the manufacturer’s instructions. Subtraction hybridization was conducted to perform subtraction in both forward and reverse directions. After two rounds of hybridization, PCR was conducted to selectively amplify cDNAs differentially expressed between ORFV-inoculated and normal MDBK cells. Each PCR amplification described below was conducted using a thermal cycler MyCycler (Bio-Rad) in a total volume of 25 μL with 1 μL template DNA, 0.5 U of 50 × Advantage cDNA polymerase (Clontech, USA), 200 μM of each dNTP, and forward and reverse primers (1 μM each). The cDNA fragments for subtracted secondary PCR products were inserted into the pMD18-T vector (TaKaRa, Dalian, China) with T4 DNA ligase and transformed into DH5a *Escherichia coli* cells. The clones with recombinant plasmid were identified by the LB-ampicillin/IPTG/X-Gal medium colony screening. Recombinant white clones were selected randomly and amplified by PCR using the M13 primer to construct the corresponding SSH cDNA library. The clones that yielded a single PCR product were selected for the next analysis.

### qRT-PCR analysis of CypB expression in ORFV-infected MDBK cells

Among the differentially expressed genes obtained by SSH technique, CypB was found to be significantly up-regulated in the ORFV-infected MDBK cells at an early phase of infection. The qRT-PCR method based on the CypB gene was applied to determine the mRNA expression level of CypB in MDBK cells infected with ORFV-Jilin (MOI = 3) for 0 h, 2 h, 4 h, 8 h, 12 h, 18 h and 24 h. The primer sets used in the study were specific for CypB (forward primer 5′-GAGACGGCACTGGAGGTAAG-3′; reverse primer 5′-TCGTGATGAAGAACTGGGAG-3′). Briefly, total RNA was isolated from both mock- and ORFV-inoculated cell lysates using RNAiso Plus (Takara, Dalian, China) according to the manufacturer’s instructions. Then, 200 ng of each RNA sample was used in a Oligo (dT) cDNA synthesis. The qPCRs for CypB gene were performed in 20 μL of reaction mixture containing 10 μL SYBR Premix ExTaq (Takara, Dalian, China), 0.2 μM of each oligonucleotide primer, 100 ng of each cDNA sample, and 7.2 μL ddH_2_O. Each sample was repeated three times. Fluorescent signals were analyzed by an ABI PRISM 7000 (Applied Biosystems, Tokyo, Japan). Data were statistically analyzed by Student’s *t*-test using GraphPad Prism 6 software.

### Western blotting analysis of CypB expression in ORFV-infected MDBK cells

The MDBK cells at hours 0, 2, 4, 8, 12, 18 and 24 post infection were washed thoroughly and lysed in cell lysis buffer (20 mM Tris [pH 7.5], 150 mM NaCl, 1% Triton X-100, sodium pyrophosphate, β-glycerophosphate, EDTA, Na3VO4, leupeptin). The lysates were briefly sonicated and cleared by centrifugation for 5 min at 12,000 g at 4 °C. The lysates were further denatured by incubation for 10 min at 95 °C in sample buffer (2% SDS, 10% glycerol, 50 mM Tris [pH 6.8], 5% β-mercaptoethanol, 0.01% bromophenol blue). The samples were then subjected to sodium dodecyl sulfate-polyacrylamide gel electrophoresis (SDS-PAGE) and transferred to polyvinylidene fluoride membranes (Millipore, Bedford, MA). After incubation in blocking buffer (5% nonfat milk powder in PBS) for 1 h at room temperature, the membrane was reacted with anti-Cyclophilin B antibody (1:1000) overnight at 4 °C and HRP-conjugated secondary antibodies (1:3000) for 1 h at 37 °C. The antibody-antigen complex was visualized by enhanced chemiluminescence (ECL) (Thermo Fisher Scientific).

### Anti-ORFV activity of CsA in vitro detected by MTT assay

MTT assay used for measuring cell survival and proliferation was performed in order to determine the effect of CsA on the inhibitory ratio of ORFV. Briefly, a confluent monolayer of MDBK cells grown on 96-well plates were inocubated with ORFV-Jilin (MOI = 0.1) and incubated in a CO_2_ incubator with a 5% CO_2_ atmosphere. After 2 h of adsorption, the maintenance medium containing various concentrations of CsA (0.05, 0.1, 0.2, 0.4, 0.8, 1.6, 3.2 μg/mL) was added after removing the virus inoculum. At 24 h after drug action, 100 μL MTT (tetrazolium salt 3-[4,5-dimethylthiazol-2-yl]-2,5-diphenyltetrazolium bromide) were added to each well and kept in a dark environment for 4 h at 37 °C. Then, MTT was aspirated and 100 μL well^−1^ of DMSO (Merck, Darmstad, Germany) was added to each well. Subsequently, the absorbance value at a wavelength of 570 nm was measured using a UV–visible spectrophotometer (LPB Pharmacia, Bromma, Sweden) for the calculating the inhibitory rate of CsA on ORFV proliferation. The untreated cell cultures were cultured under the same conditions. Data were statistically analyzed by one-way ANOVA followed by Fisher’s least significant difference (LSD) test using GraphPad Prism 6 software.

### CypB gene silencing by siRNA

Based on the sequences of CypB, two pairs of siRNAs, CypB-specific siRNA and negative siRNA control (scrambled siRNA) were designed and synthesized by Shanghai GenePharma Co., Ltd. The sequences are shown in Table 1. The cells were grown on 12-well plates and incubated in a CO_2_ incubator with a 5% CO_2_ atmosphere. Approximately 50–60% confluent, the cells were transfected with 35 nM siRNA by using X-tremeGENE siRNA transfection reagent (Roche, USA) according to the manufacturer’s protocol. The transfected cells were then washed with MEM without serum and incubated in MEM supplemented with 2% fetal bovine serum. Untransfected MDBK cells were used as a control. Typical siRNA transfection efficiency was found to be 83% for MDBK cells as monitored by fluorescein-labeled control siRNA duplex. CypB knockdown was confirmed 48 h post siRNA transfection by qRT-PCR and Western blotting (data not shown).

**Table 1:** List of siRNA sequences used in this study

Name	Primer Sequences (5′-3′)	
CypB specific siRNA	up	GCAUCUACGGUGAACGCUUTT
	down	AAGCGUUCACCGUAGAUGCTT
Negative control siRNA	up	UUCUCCGAACGUGUCACGUTT
	down	ACGUGACACGUUCGGAGAATT

### Immunofluorescence assay after siRNA knockdown of CypB

The MDBK cells were mock-transfected or transfected with CypB siRNAs or scrambled siRNAs for 48 h and subjected to immunofluorescence assay. Cells cultured on glass slides were washed gently in phosphate buffered saline (PBS), and fixed with 4% paraformaldehyde for 30 min, PBS washed (three times, each 5 min); 0.2% Triton X-100 enhanced the permeability of cell membranes for 10 min at room temperature and PBS washed; 5% skim milk incubated 1 h, PBS washed. Then cells were incubated with PE-Cy5 conjugated rabbit anti-Cyclophilin B antibody (1:500) at 4 °C over the night. Cell nuclei were stained blue with DAPI for 15 min. Finally, the samples were washed three times with PBS and observed under immunofluorescent microscope.

### Effect of CypB gene silencing on ORFV replication

At 48 h post siRNA transfection, the siRNA-treated cells were infected with ORFV-Jilin (MOI = 3), respectively. Viral DNA was extracted from cell cultures at 12 h, 24 h, 36 h, 48 h, 60 h and 72 h postinfection. The qPCR method based on the DNA polymerase gene of ORFV was applied to determine the viral DNA copy number. The qPCR assay was performed as described previously [15]. Furthermore, viral yields were quantitated in the siRNA-treated cells by the 50% tissue culture infectious dose (TCID_50_) method. Briefly, the siRNA-treated cells were infected with 100 TCID_50_ (10^6.5^) of ORFV, and the infection was allowed to proceed for the indicated time periods. Untransfected MDBK cells were used as a control. Data were statistically analyzed by one-way ANOVA followed by Fisher’s LSD test using GraphPad Prism 6 software.

### Statistical analysis

Statistical analysis was carried out using GraphPad Prism v6.0 (GraphPad Software, La Jolla, CA). Student’s *t*-test and one-way ANOVA followed by Fisher’s LSD test were used for comparisons of two and multiple groups, respectively. Data were expressed as the means with error bars depicting ± SEM, and differences were considered statistically significant at * *P* < 0.05, ** *P* < 0.01, *** *P* < 0.001.

## Results

### CypB was found to be significantly up-regulated in the ORFV-infected MDBK cells by SSH technique

In order to explore host genes inovolved in the process of ORFV invasion, the differentially expressed genes from ORFV-infected MDBK cells at an early phase of infection were identified using SSH technique. A total of 81 expressed sequence tags (ESTs) were obtained from the SSH libraries by sequencing and alignment. Among of all the ESTs, 60 ESTs came from the forward SSH library and 21 ESTs came from the reverse SSH library. BLAST analysis against the Genbank database revealed that 79 ESTs showed significantly homology to known genes. Of those, all the ESTs showed significantly homology with *Bos Taurus* related sequences (Tables 2 and 3). CypB as an important member of cyclophilins family, was found to be significantly up-regulated in the ORFV-infected MDBK cells at an early phase of infection.

**Table 2:** Similarity between expressed sequence tags in the forward suppression subtractive hybridization library and genes in GenBank

EST No.	Species	Gene homology	Gene name	Fold upregulation
F-1	*Bos Taurus*	Bos taurus beta-actin (ACTB) mRNA	ACTB	1.5
F-2	*Bos Taurus*	Bos taurus actin, gamma 1 (ACTG1), mRNA	ACTG1	1.7
F-3	unknown	unknown	unknown	3.4
F-4	*Bos Taurus*	Bos taurus vacuole membrane protein 1 (VMP1), mRNA	VMP1	3.7
F-5	*Bos Taurus*	Bos taurus ring finger protein 19B (RNF19B), mRNA	RNF19B	1.9
F-6	*Bos Taurus*	Bos taurus zinc finger protein 408 (ZNF408), mRNA	ZNF408	2.3
F-7	*Bos Taurus*	Bos taurus zinc finger CCCH-type containing 14 (ZC3H14), transcript variant 1, mRNA	ZC3H14	1.5
F-8	*Bos Taurus*	Bos taurus ATPase, Na+/K+ transporting, beta 1 polypeptide (ATP1B1), mRNA.	ATP1B1	3.4
F-9	*Bos Taurus*	Bos taurus poly-U binding splicing factor 60KDa (PUF60), mRNA.	PUF60	2.3
F-10	unknown	unknown	unknown	2.6
F-11	*Bos Taurus*	Bos taurus eukaryotic translation initiation factor 3, subunit L (EIF3L), mRNA	EIF3L	3.7
F-12	*Bos Taurus*	Bos taurus eukaryotic translation elongation factor 1 alpha 1 EEF1A1), mRNA	EEF1A1	3.4
F-13	*Bos Taurus*	Bos taurus eukaryotic translation initiation factor 5 (EIF5), mRNA	EIF5	2.9
F-14	*Bos Taurus*	Bos taurus cytohesin 2 (CYTH2), mRNA	CYTH2	1.6
F-15	*Bos Taurus*	Bos taurus transmembrane emp24 protein transport domain containing 1 (TMED1), mRNA	TMED1	3.6
F-16	*Bos Taurus*	Bos taurus transmembrane protein 179B (TMEM179B), mRNA	TMEM179B	2.8
F-17	*Bos Taurus*	Bos taurus neuropilin 2 (NRP2), mRNA	NRP2	2.1
F-18	*Bos Taurus*	Sin3A-associated protein, 18 kDa (SAP18), mRNA	SAP18	2.4
F-19	*Bos Taurus*	Bos taurus renin binding protein (RENBP), mRNA	RENBP	1.3
F-20	*Bos Taurus*	Bos taurus signal sequence receptor, alpha (SSR1), mRNA	SSR1	1.9
F-21	*Bos Taurus*	Bos taurus mitogen-activated protein kinase kinase 6 (MAP2K6), mRNA	MAP2K6/MKK6	9.2
F-22	*Bos Taurus*	Bos Taurus solute carrier family 1 (neuronal/epithelial high affinity glutamate transporter, system Xag), member 1 (SLC1A1), mRNA.	SLC1A1	2.7
F-23	*Bos Taurus*	Bos taurus isolate Mcg489 mitochondrion, complete genome	Mcg489 mitochondrion	2.5
F-24	*Bos Taurus*	Calmodulin 1 (phosphorylase kinase, delta) (CALM1), mRNA	CALM1	2.3
F-25	*Bos Taurus*	Bos taurus enhancer of rudimentary homolog (Drosophila) (ERH), mRNA	ERH	1.7
F-26	*Bos Taurus*	Bos taurus threonyl-tRNA synthetase-like 1 (TARSL1), mRNA, complete cds.	TARSL1	2.1
F-27	*Bos Taurus*	Bos taurus threonyl-tRNA synthetase 2, mitochondrial (putative) (TARS2), mRNA	TARS2	1.8
F-28	*Bos Taurus*	Bos taurus S100 calcium binding protein A2 (S100A2), mRNA	S100A2	9.7
F-29	*Bos Taurus*	Bos taurus lactate dehydrogenase B (LDHB), mRNA, complete cds.	LDHB	3.5
F-30	*Bos Taurus*	Bos taurus H3 histone, family 3A (H3F3A), mRNA.	H3F3A	4.2
F-31	*Bos Taurus*	Bos taurus heterogeneous nuclear ribonucleoprotein U (scaffold attachment factor A) (HNRNPU), mRNA.	HNRNPU	2.9
F-32	*Bos Taurus*	Bos taurus RAB2A, member RAS oncogene family (RAB2A), mRNA.	RAB2A	1.5
F-33	*Bos Taurus*	Bos taurus alanyl-tRNA synthetase (AARS), mRNA	AARS	1.9
F-34	*Bos Taurus*	Bos taurus minichromosome maintenance complex component 4 (MCM4), mRNA	MCM4	2.7
F-35	*Bos Taurus*	Bos taurus tubulin, beta 6, mRNA	TUBB6	1.3
F-36	*Bos Taurus*	Bos taurus tubulin, alpha 1c (TUBA1C), mRNA	TUBA1C	1.7
F-37	*Bos Taurus*	Bos taurus tubulin, alpha 1b (TUBA1B), mRNA	TUBA1B	1.2
F-38	*Bos Taurus*	Bos taurus tropomyosin 1 (alpha) (TPM1), mRNA	TPM1	2.0
F-39	*Bos Taurus*	Bos taurus tropomyosin 4 (TPM4), mRNA	TPM4	1.5
F-40	*Bos Taurus*	Bos taurus minichromosome maintenance complex component 6 (MCM6), mRNA	MCM6	2.3
F-41	*Bos Taurus*	Bos taurus chromosome 10 open reading frame, human C14orf1 (C10H14orf1), mRNA.	C10H14orf1	2.4
F-42	*Bos Taurus*	Bos taurus CLPTM1-like (CLPTM1L), mRNA	CLPTM1L	4.3
F-43	*Bos Taurus*	Bos taurus CD151 molecule (Raph blood group) (CD151), mRNA	CD151	7.6
F-44	*Bos Taurus*	Bos taurus ubiquinol-cytochrome c reductase binding protein (UQCRB), mRNA.	UQCRB	2.6
F-45	*Bos Taurus*	Bos taurus low molecular mass ubiquinone-binding protein mRNA, complete cds.	Low molecular mass ubiquinone-binding protein	2.1
F-46	*Bos Taurus*	Bos taurus dynein, cytoplasmic 1, light intermediate chain 1 (DYNC1LI1), mRNA	DYNC1LI1	3.5
F-47	*Bos Taurus*	Bos taurus glucose-6-phosphate isomerase (GPI), mRNA	GPI	2.9
F-48	*Bos Taurus*	Bos taurus galactosidase, beta 1 (GLB1), mRNA	GLB1	2.4
F-49	*Bos Taurus*	Bos taurus proteasome (prosome, macropain) activator subunit 1 (PA28 alpha) (PSME1), mRNA.	PSME1	1.3
F-50	*Bos Taurus*	Bos taurus peptidylprolyl isomerase B (cyclophilin B) (PPIB), mRNA	PPIB	10.2
F-51	*Bos Taurus*	Bos taurus peroxiredoxin 2 (PRDX2), mRNA.	PRDX2	3.4
F-52	*Bos Taurus*	Bos taurus proteolipid protein 2 (colonic epithelium-enriched) (PLP2), mRNA.	PLP2	3.7
F-53	*Bos Taurus*	Bos taurus voucher proven bull 27,223 mitochondrion, complete genome.	Bos taurus voucher proven bull 27,223 mitochondrion	2.1
F-54	*Bos Taurus*	Bos taurus Fas-activated serine/threonine kinase (FASTK), mRNA.	FASTK	4.6
F-55	*Bos Taurus*	Bos taurus ferritin, light polypeptide (FTL), mRNA	FTL	2.5
F-56	*Bos Taurus*	Bos taurus Finkel-Biskis-Reilly murine sarcoma virus (FBR-MuSV) ubiquitously expressed (FAU), mRNA.	FAU	1.2
F-57	*Bos Taurus*	Bos taurus acyl-CoA thioesterase 7 (ACOT7), mRNA	ACOT7	1.9
F-58	*Bos Taurus*	Bos taurus adenine phosphoribosyltransferase (APRT), mRNA	APRT	1.5
F-59	*Bos Taurus*	Bos taurus mannosyl (alpha-1,3-)-glycoprotein beta-1,2-N-acetylglucosaminyltransferase (MGAT1), mRNA, complete cds	MGAT1	1.7
F-60	*Bos Taurus*	Bos taurus lactate dehydrogenase C (LDHC), transcript variant 1, mRNA.	LDHC	2.3

**Table 3:** Similarity between expressed sequence tags in the rverse suppression subtractive hybridization library and genes in GenBank

EST No.	Species	Gene homology	Gene name	Fold downregulation
R-1	*Bos Taurus*	Bos taurus mitogen-activated protein kinase kinase 4 (MAP2K4), mRNA.	MAP2K4/MKK4	8.7
R-2	*Bos Taurus*	Bos taurus neuroplastin (NPTN), mRNA	NPTN	1.9
R-3	*Bos Taurus*	Bos taurus nuclear transport factor 2 (NUTF2), mRNA	NUTF2	2.5
R-4	*Bos Taurus*	Bos taurus S100 calcium binding protein A4 (S100A4), mRNA	S100A4	9.6
R-5	*Bos Taurus*	Bos taurus acyl-CoA synthetase long-chain family member 5 (ACSL5), mRNA, complete cds	ACSL5	2.9
R-6	*Bos Taurus*	Bos taurus eukaryotic translation elongation factor 2 (EEF2), mRNA	EEF2	3.8
R-7	*Bos Taurus*	Bos taurus poly-U binding splicing factor 60KDa (PUF60), mRNA	PUF60	1.3
R-8	*Bos Taurus*	Bos taurus H3 histone, family 3B (H3.3B) (H3F3B), mRNA	H3F3B	2.6
R-9	*Bos Taurus*	Bos taurus glyceraldehyde-3-phosphate dehydrogenase (GAPDH), mRNA	GAPDH	1.2
R-10	*Bos Taurus*	Bos taurus NADH dehydrogenase (ubiquinone) complex I, assembly factor 1 (NDUFAF1), mRNA	NDUFAF1	1.7
R-11	*Bos Taurus*	Bos taurus solute carrier family 51, beta subunit (SLC51B), mRNA	SLC51B	3.2
R-12	*Bos Taurus*	Bos taurus ferredoxin 1 (FDX1), mRNA	FDX1	2.5
R-13	*Bos Taurus*	Bos taurus transforming growth factor beta 1 induced transcript 1 (TGFB1I1), mRNA	TGFB1I1	1.4
R-14	*Bos Taurus*	Bos taurus heat shock 70 kDa protein 8 (HSPA8), mRNA	HSPA8	10.2
R-15	*Bos Taurus*	Bos taurus vimentin (VIM), mRNA	VIM	6.4
R-16	*Bos Taurus*	Bos taurus lectin, galactoside-binding, soluble, 1 (LGALS1), mRNA	LGALS1	2.1
R-17	*Bos Taurus*	Bos taurus clone IMAGE:7,961,517 NDP kinase NBR-A mRNA, complete cds	NDP kinase NBR-A	1.5
R-18	*Bos Taurus*	Bos taurus polymerase (RNA) I polypeptide D (POLR1D), mRNA	POLR1D	1.5
R-19	*Bos Taurus*	Bos taurus guanine nucleotide binding protein (G protein), alpha inhibiting activity polypeptide 1 (GNAI1), mRNA	GNAI1	1.2
R-20	*Bos Taurus*	Bos taurus annexin A2 (ANXA2), mRNA	ANXA2	1.9
R-21	*Bos Taurus*	Bos taurus Williams Beuren syndrome chromosome region 22 (WBSCR22), mRNA	WBSCR22	2.3

### Verification of the mRNA expression levels of CypB gene by qRT-PCR

**Fig. 1:**
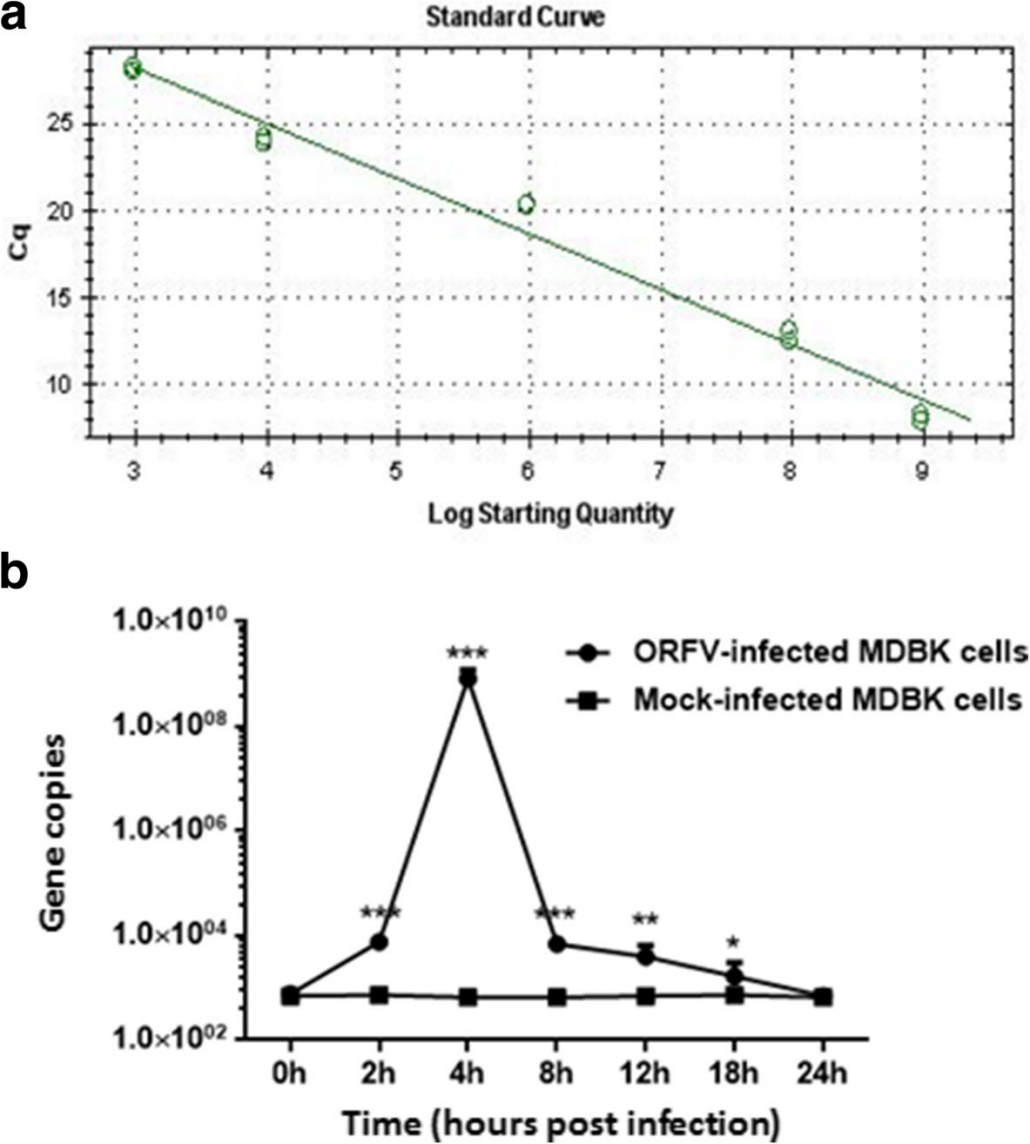
Verification of the mRNA expression levels of CypB gene identified from the forward SSH library by qRT-PCR. **a** According to qRT-PCR, the standard curve established showed an efficiency of 106.7% with R^2^ of 0.991. **b** The mRNA expression levels of CypB gene in mock- and ORFV-infected MDBK cells at 0, 2, 4, 8, 12, 18 and 24 h post infection were measured by qRT-PCR. Statistical analysis was performed by Student’s *t*-test using GraphPad Prism 6 software. Values are the means ± SEM. (* *P* < 0.05, ** *P* < 0.01, *** *P* < 0.001). As shown in Fig. 1b, the transcript levels of CypB was overexpressed in MDBK cells infected with ORFV-Jilin (MOI = 3) compared with mock-infected MDBK cells, with CypB mRNA levels 10^6^-fold higher in ORFV-infected MDBK cells at 4 h post infection than in the corresponding mock-infected MDBK cells

We further verified the mRNA expression levels of CypB gene by qRT-PCR (Fig. 1a). CypB was confirmed by qRT-PCR as differentially expressed. The transcript levels of CypB was overexpressed in MDBK cells infected with ORFV-Jilin compared with mock-infected MDBK cells (Fig. 1b), with CypB mRNA levels 10^6^-fold higher in ORFV-infected MDBK cells at 4 h post infection than in the corresponding mock-infected MDBK cells.

### Verification of the protein expression levels of CypB by Western blotting

**Fig. 2:**
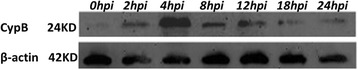
Verification of the protein expression levels of CypB gene identified from the forward SSH library with western blotting. The MDBK cells at hours 0, 2, 4, 8, 12, 18 and 24 post infection were collected and lysed in cell lysis buffer (20 mM Tris [pH 7.5], 150 mM NaCl, 1% Triton X-100, sodium pyrophosphate, β-glycerophosphate, EDTA, Na3VO4, leupeptin). The lysates were subjected to SDS/PAGE and Western blot analysis with anti-Cyclophilin B antibody (diluted with 1:1000). β-actin was used as an internal control. An increased expression of CypB gene was observed in the ORFV-infected MDBK cells, especially at 4 h post infection. There were no obvious changes observed in the levels of β-actin

The expression of CypB gene was confirmed by Western blotting using β-actin as an internal control. Western blot analysis showed increased expression in the ORFV-infected MDBK cells, especially at 4 h post infection (Fig. 2). The results were consistent with those obtained from qRT-PCR. These findings suggested that changes in the expression of CypB gene and protein are associated with ORFV early infection.

### Inhibition effect of CsA on ORFV proliferation in MDBK cells

**Fig. 3:**
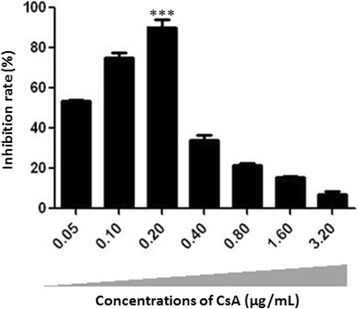
Inhibition effect of CsA on ORFV proliferation in MDBK cells. The MDBK cells infected with ORFV (MOI = 0.1) were exposed to increasing concentrations of CsA (0.05, 0.1, 0.2, 0.4, 0.8, 1.6, 3.2 μg/mL) and maximal ethanol concentration used to solubilize and dilute CsA as vehicle. The anti-ORFV activity of CsA in vitro was detected by MTT assay. Subsequently, the absorbance value at a wavelength of 570 nm was measured for the calculating the inhibitory rate of CsA on ORFV proliferation. The untreated cells were cultured under the same conditions. Data were statistically analyzed by one-way ANOVA followed by Fisher’s LSD test using GraphPad Prism 6 software. Values are the means ± SEM. (*** *P* < 0.001). The results showed that treatment with 0.2 μg/mL CsA could effectively inhibit the cytotoxicity induced by ORFV

The inhibition of CsA on ORFV proliferation in vitro was evaluated by MTT assay. The MDBK cells infected with ORFV were exposed to increasing concentrations of CsA from 0 to 3.2 μg/mL and maximal ethanol concentration used to solubilize and dilute CsA as vehicle. As demonstrated in Fig. 3, the inhibition rate was increased in different concentrations of CsA (0.05, 0.1, 0.2, 0.4, 0.8, 1.6, 3.2 μg/mL) in comparision with the control (no treatment) and this increase was significant in 0.2 μg/mL (*** *P* < 0.001). Our results showed that treatment with 0.2 μg/mL CsA could effectively inhibit the cytotoxicity induced by ORFV.

### Examination of siRNA effect by Immunofluorescence assay

**Fig. 4:**
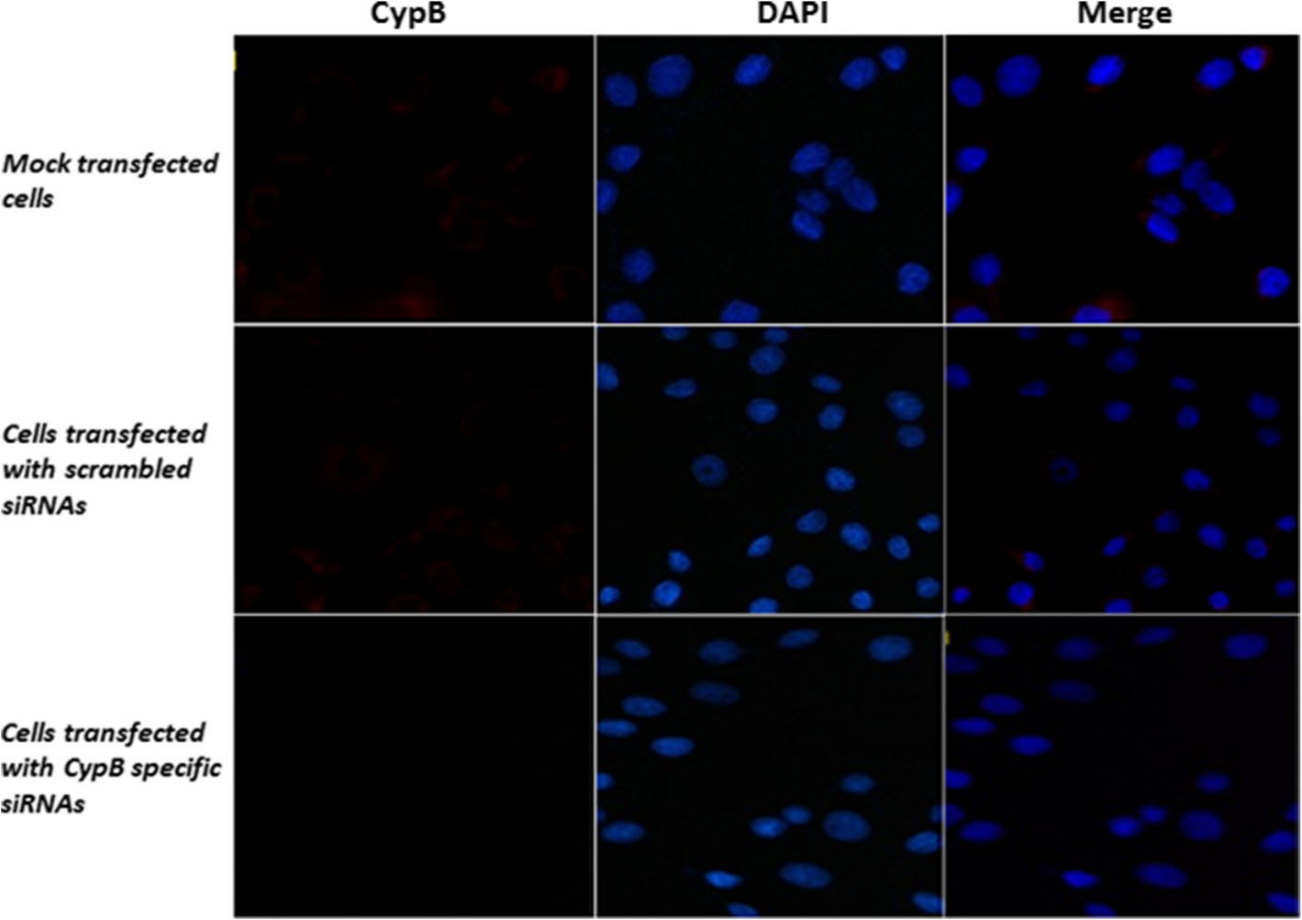
Examination of siRNA effect by Immunofluorescence assay. The MDBK cells were mock-transfected or transfected with CypB siRNAs or scrambled siRNAs for 48 h and subjected to immunofluorescence assay. As shown in Fig. 4, the CypB-specific red fluorescence signals distributed in the cytoplasm of MDBK cells were found in the cells transfected with scrambled siRNAs, which was similar to that in mock-transfected cells. However, no red fluorescence signals were observed in the cells transfected with CypB specific siRNAs. Nuclei were stained *blue* with DAPI

The immunofluorescence assay was performed in order to determine the inhibitory effect of CypB specific siRNAs. As shown in Fig. 4, the CypB-specific red fluorescence signals distributed in the cytoplasm of MDBK cells was found to be attenuated at 48 h post transfection. The cells transfected with scrambled siRNAs exhibited relative apparent fluorescent signals, which was similar to that in mock-transfected cells. However, no red fluorescence signals were observed in the cells transfected with CypB specific siRNAs. Nuclei were stained blue with DAPI.

### Examination of siRNA effect by qPCR

**Fig. 5:**
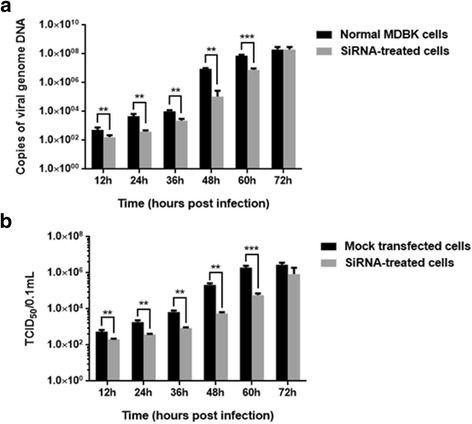
The inhibitory effect of of CypB specific siRNAs on ORFV replication in MDBK cells. **a** At 48 h post siRNA transfection, the siRNA-treated cells were infected with ORFV-Jilin, respectively. Viral DNA was extracted from cell cultures at 12, 24, 36, 48, 60 and 72 h postinfection. The qPCR method based on the DNA polymerase gene of ORFV was applied to determine the viral DNA copy number. **b** Viral yields were quantitated in the siRNA-treated cells by TCID_50_ assay. TCID_50_ values are the means of three repeat titrations at the same points indicated. Statistical analysis was performed using one-way ANOVA followed by Fisher’s LSD test. Values are the means ± SEM. (** *P* < 0.01, *** *P* < 0.001)

To quantify the effect of siRNA on viral replication, the viral genome copy number was determined at 12 h, 24 h, 36 h, 48 h, 60 h and 72 h postinfection by qPCR based on the DNA polymerase gene of ORFV. The results indicated a marked reduction in the relative expression level of viral genome copies, compared to the mock group at 12 h, 24 h, 36 h, 48 h, 60 h and 72 h postinfection, when the CypB specific siRNA were used. At 48 h postinfection, the number of viral genome copies of the siRNA-treated cells was only 1 % in the ORFV-infected cells (Fig. 5a).

### Examination of siRNA effect by infectious virus assay

To further investigate the inhibitory effect of siRNAs, the microtiter method was used for titration of ORFV at 5 days postinfection. The results (Fig. 5b) showed that viral titers in untransfected MDBK cells was higher than in the siRNA-treated cells at 12 h, 24 h, 36 h, 48 h, 60 h and 72 h postinfection. In untransfected MDBK cells, titers reached a peak of around 1 × 10^6.3^/0.1 mL (TCID_50_) at 60 h postinfection, however, titers reached similar values in the siRNA-treated cells at 72 h postinfection.

## Discussion

Viruses are strict intracellular pathogens that require the cellular environment to complete a successful infection. During the infection process, viruses could affect several major cellular responses by altering the expression of the key host genes. Considering viral infection strongly relies on several factors from the host, the study of virus-host interactions is essential to the understanding of the infection process of disease and the mechanism of the virus persistence in the host, and to help with the development of effective vaccines and perhaps the cure of viral infections.

ORFV is a highly epitheliotropic virus, that infects damaged or scarified skin and replicates in regenerating epidermal keratinocytes. An important characteristic of the virus is its ability to cause repeated and persistent infections [16] even in the presence of an intensive inflammatory and multiple host immune response [3, 17]. At present, little is known about the mechanisms of ORFV infection of host, although several studies have demonstrated that ORFV encoded protein might interact with a host protein involved in the pathway and/or might mimic the structurally similar host protein to invade/manipulate the cellular pathway [18, 19].

With the purpose of identify genes involved in ORFV early invasion, we constructed a cDNA library and identified the differently expressed genes from ORFV-infected MDBK cells at an early phase of infection in the study. By using SSH technique, a total of 81 EST sequences with differing functions were identified, including 60 differently up-regulated genes from the forward library and 21 differently down-regulated genes from the reverse library. CypB as an important member of cyclophilins family, was found to be significantly up-regulated in the ORFV-infected MDBK cells at an early phase of infection. In the present study, the upregulation of CypB in host cells was further validated by qRT-PCR and Western blotting. The results showed that CypB was overexpressed in MDBK cells infected with ORFV-Jilin at 4 h post infection, which were consistent with those obtained by SSH technique.

Recently, increasing studies have demonstrated that cellular CypB are closely related with viral infections such as JEV [11], HCV [12] and HPV 16 [13]. However, the role of CypB in ORFV proliferation have remained unknown. By using CsA, we determined that it could effectively inhibit the cytotoxicity induced by ORFV, which suggested that CypB was required for the efficient proliferation of ORFV in vitro. To further ascertain the regulatory role of CypB for ORFV replication, CypB specific siRNAs were synthesized and then transfected. The efficiency of inhibition of viral replication was evaluated by immunofluorescence assay, qPCR and infectious virus assay. The results demonstrated that silencing of CypB gene by RNAi could efficiently inhibit ORFV genome replication and infectious virus production. On the basis of the above studies, CypB has been confirmed to facilitate ORFV replication.

Moreover, CypB has been demonstrated to involve in inflammatory events [20]. The overexpressed CypB could activate the extracellular signal-regulated kinase intracellular signaling pathway such as ERK signaling through binding with CD147, which has been shown to function as a signalling receptor for CypB to mediate chemotactic activity towards a variety of immune cells [21–23]. Currently, the upregulation of CypB has been confirmed to facilitate ORFV replication, presumably through interactions with CD147 or other some agents to activate the associated immunological and inflammatory signaling pathways. Further investigations on the cooperation between these molecules might provide new insights on the mechanisms controlling ORFV infection within target cells and lead to the development of new therapeutic tools to block the virus infection.

## Conclusions

The results of the present study demonstrated that CypB could effectively facilitate the replication of ORFV, suggesting CypB might be involved in the process of ORFV infection. However, the detailed interaction between CypB and ORFV is still unknown. Further studies will be focused on the interplay between ORFV and cellular CypB, which may contribute to clarify virus pathogenesis and the development of antiviral vaccine candidates and novel inhibitors.

## Abbreviations

CsA: Cyclosporin A
CypB: Cyclophilin B
ESTs: Expressed sequence tags
EtBr: Ethidium bromide
FBS: Fetal bovine serum
HCV: Hepatitis C virus
HPV 16: Human papillomavirus type 16
JEV: Japanese encephalitis virus
MDBK: Madin―Darby bovine kidney cells
MEM: Minimal essential medium
MOI: Multiplicity of infection
mRNA: Messenger RNA
MTT: Tetrazolium salt 3-[4,5-dimethylthiazol-2-yl]-2,5-diphenyltetrazolium bromide
ORFV: Orf virus
PBS: Phosphate buffered saline.
PPIase: Peptidyl-prolyl *cis-trans* isomerase
RNAi: RNA interference
SDS-PAGE: Sodium dodecyl sulfate-polyacrylamide gel electrophoresis
TCID_50_: 50% tissue culture infectious dose
